# Unbiased inference for echocardiogram urgency prediction using double machine learning

**DOI:** 10.1371/journal.pone.0338922

**Published:** 2026-01-07

**Authors:** Yiqun Jiang, Wenli Zhang, Yu-Li Huang, Cameron MacKenzie, Qing Li

**Affiliations:** 1 Department of Industrial and Manufacturing Systems Engineering, Iowa State University, Ames, Iowa, United States of America; 2 Department of Information Systems and Business Analytics, Iowa State University, Ames, Iowa, United States of America; 3 Robert D. and Patricia E. Kern Center for the Science of Health Care Delivery, Mayo Clinic, Rochester, Minnesota, United States of America; Scuola Superiore Sant'Anna, ITALY

## Abstract

The increased utilization of echocardiography in clinical practice has witnessed a substantial rise, underscoring its pivotal role as a diagnostic tool for various cardiovascular conditions. However, due to the relative scarcity of echocardiography tests, challenges persist in efficiently prioritizing patients for echocardiographic assessments. In this study, we develop a model to assess the urgency of appointments by considering both clinical and administrative variables extracted from Electronic Health Record data. We use double machine learning techniques to analyze these variables and improve our predictions of patient urgency. Traditional methods for estimating variable effects have limitations, particularly in our research context, where clinical and administrative variables may influence one another while also directly impacting the outcome (i.e., the urgency of appointments). In this work, we address this issue by developing an urgency stratification model using double machine learning, which disentangles the complex relationships between variables. Our evaluations demonstrate that the proposed model not only outperforms traditional machine learning methods in predicting appointment urgency but also provides robust estimations of variable effects. Specifically, our results underscore the critical roles of *administrative* variables and cancer-related *comorbidity* variables in patient prioritization and appointment urgency prediction. By leveraging double machine learning techniques, our method can enhance the efficiency and effectiveness of echocardiography utilization in clinical practice. It provides clinicians with actionable insights for patient prioritization, facilitating the timely identification of urgent cases and the optimal allocation of resources. Our work contributes to the advancement of healthcare practices by leveraging sophisticated analytics to improve patient care delivery and streamline clinical workflows in echocardiography laboratories. A similar research design can also be extended to other advanced yet limited laboratory tests to help prioritize medical resources.

## Introduction

The echocardiography laboratory is a crucial and multi-faceted procedure area that provides management-defining cardiac diagnostics for a wide spectrum of patients. Echocardiography is the primary diagnostic examination for various pericardial syndromes, such as acute pericarditis, recurrent pericarditis, and constrictive pericarditis [1]. Research finds echocardiography has emerged as a crucial tool in the reduction of mortality rates attributed to cardiovascular diseases over the last four decades [2]. From 2001 to 2011, there is a consistent rise in the total number and occurrence rate of echocardiography, with average annual growth rates of 3.41% and 3.04%, respectively [2]. However, given the increasing demand for echocardiography and its limited availability, there remains opportunities to prioritize echocardiography tests for urgent patients [2].

Specifically, in this study, we collaborated with a large multispecialty hospital and medical facility, where the echocardiogram laboratory performs complex diagnostic cardiac imaging for over 200 outpatients daily in Rochester, Minnesota. In the past years, the echocardiogram laboratory unfortunately has experienced access challenges in the outpatient practice as a result of limited sonographer/physician FTEs, increased demands, escalating complexity of patients, and the pressure of staying abreast of advanced technology. In response, the medical facility we collaborated with implemented several pilot solutions, utilizing process mapping techniques to redefine different patient care processes to increase efficiency, teamwork, and workload distribution. However, without employing a comprehensive, data-driven, and algorithmic approach that incorporates all relevant clinical and administrative variables, the improvement remained modest.

The need for prioritizing patients based on the urgency of their medical tests or procedures has been widely discussed in the literature, particularly in contexts such as pandemic testing (e.g., COVID-19) [3], emergency department lab tests [4], and genetic testing [5]. However, while these studies provide valuable insights into general prioritization strategies, they primarily address conditions with relatively well-defined urgent criteria and high-throughput testing systems. In contrast to these existing works, the prioritization of echocardiograms presents unique challenges that have not been extensively addressed in the literature.

In this context, the real-world impact of this study is significant. By introducing a data-driven, predictive model for urgency stratification, we aim to improve the prioritization of patients who require timely echocardiography. This model will help optimize clinical resources and reduce delays in diagnosing and treating life-threatening conditions. Moreover, by better aligning appointments with clinical urgency, the model could enhance patient outcomes and increase overall healthcare system efficiency.

In our research context, analyzing all types of variables is crucial due to the complexity of patient backgrounds: patients are often referred from various medical and surgical specialties and typically present with medical complexities, diverse demographics, and varying pathologies. This heterogeneity introduces significant variability in clinical scenarios, with cases spanning from straightforward and routine to highly intricate and urgent. For instance, some patients may require routine follow-ups for chronic conditions, while others may present with acute, life-threatening pathologies necessitating immediate intervention. However, in practice, our collaborators often schedule many cases into similar time slots following a first-come, first-served policy. It results in overbooking of the echocardiogram laboratory, as it aims to accommodate all requests equally: this practice increases wait times of urgent patients and contributes to dissatisfaction among both patients and medical professionals. Moreover, the current practice imposes significant challenges on medical facilities, affecting staffing, space utilization, operating hours, and overtime expenses. Most significantly, the diminished echocardiogram laboratory capacity leads to unmet patient needs and compromised outcomes, ultimately impacting downstream medical care and causing delays in surgical procedures. Overall, it is crucial to gain a comprehensive understanding of the most significant variables among the vast amount of relevant information, including patients’ administrative records (i.e., *administration information* variables), clinical diagnoses (i.e., *comorbidity* variables), and medical histories (i.e., *referral diagnosis* variables).

In this work, we aim to estimate the effect of various variables and provide insights for patient prioritization, while also acutely predicting appointment urgency using machine learning techniques. However, traditional methods for estimating variable effects have limitations, particularly in our research context, where clinical and administrative variables may influence one another while also directly impacting the urgency of appointments. In healthcare analytics, machine learning methods have shown significant predictive performance in various applications [6]. However, traditional machine learning methods may introduce significant bias in estimating the effects of the variables of interest [7]. Such bias arises when complex variables are naively incorporated into machine learning models without untangling their interdependencies and their influence on the outcome variable. To address both limitations, double machine learning is an ideal solution: (1) it is designed to handle complex data with confounding variables, untangle the interdependencies among variables and outcomes, and is robust against potential biases; (2) it enables more accurate predictions of appointment urgency, along with precise estimates of the effects of variables in our research setting. To summarize, in this work, we construct an urgency stratification model employing double machine learning techniques with clinical and administrative variables sourced from Electronic Health Record (EHR) data to prioritize patients requiring echocardiography.

## Materials and methods

### Data description

The dataset comprises real-world data from one of the top multispecialty hospital and medical facilities, located in Minnesota, U.S. The data were collected over a one-year period in 2019, encompassing 34,293 echocardiogram appointments. The data consisted of de-identified historical records, and no information was available to identify individual participants during or after data collection. The data was accessed on January 23, 2023 for research purposes. The dataset encompasses critical patient information, including three categories: *administrative* variables, *comorbidity* variables, and *referral diagnoses* variables. *Administrative* variables including demographic profiles, medical histories, clinical settings (e.g., inpatient vs. outpatient status), past procedures, and forthcoming appointments, were fully populated in the EHR database for all patients included in the study. *Comorbidity* and *referral diagnoses* variables are extracted from EHR utilizing natural language processing techniques. They are binary indicators representing the presence (1) or absence (0) of specific medical conditions. *Comorbidity* variables encompass a spectrum of medical conditions, while *referral diagnoses* variables capture indications for previous medical evaluations and interventions. All variables exhibit complex relationships and may have interdependencies among them.

**Fig 1:**
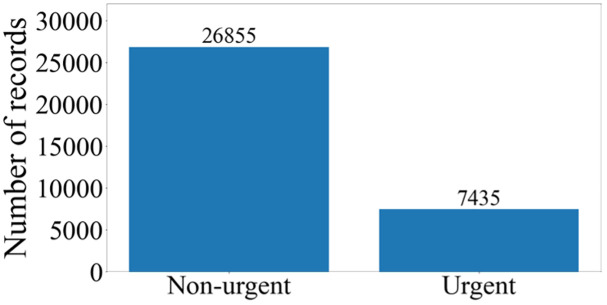
Patients’ Urgency Classification Distribution.

All variables in the dataset are categorical, ensuring compatibility for statistical analysis. To facilitate variable effect analysis, dummy variables are constructed for each variable category. The binary response variable in this investigation is determined by the temporal interval between appointment scheduling and appointment execution. Patients are classified as *urgent* if the appointment date falls within two days of the scheduling date, denoted by a response variable value of 1. Conversely, patients with appointment dates exceeding this two-day threshold receive a response variable value of 0, indicating *non-urgency*. While we acknowledge that clinical urgency can often be more complex and nuanced, a binary classification was selected due to the practical constraints of the echocardiography scheduling process at the study site. The goal of this classification was to prioritize patients within a limited time frame, ensuring that the most critical cases receive timely attention. The two-day threshold for determining urgency was chosen based on clinical input from healthcare providers at the institution, reflecting the typical practice in many healthcare settings. This threshold aligns with the operational realities of appointment scheduling, where urgent cases often need to be scheduled within 48 hours to avoid delays in patient care. We discuss this threshold in the context of existing institutional practices, which align with clinical expectations for prioritizing echocardiographic assessments. The distribution of the response variable (i.e., appointment urgency) is illustrated in Fig 1. A comprehensive summary of variables is provided in Table 1.

**Table 1:** Treatment Variable Description.

Administration	Procedure	Type of echocardiogram visit (TTE, TEE, Other)
ReferralType	Type of referral (External, Internal)
ReferredFrom	Referral origin (RST, Other, MCHS, RSTH, FLA, ZZRST, ARZ)
ReferredBy	The specialty that patient referred by it (CV, IM, Other, OB, Hosp, PED, FAM)
ReferredType	Referred type (Outpatient, Other)
NextDepartment	The department in which the appointment happened after the date the echocardiogram appointment was generated in the system (CV, non-CV)
NextLength	The number of days from the date the echocardiogram appointment was generated in the system to its following appointment
MadeBeforeEcho	Whether the next downstream appointment after echocardiogram is made before the date the echocardiogram appointment was generated in the system or not (Y, N)
GENDER	Gender (Female, Male)
surgeryYN	Whether the patient had a cardiovascular surgery within six months prior to the echocardiogram appointment date (N, Y)
SurgeryYN_After	Whether the patient had a surgery within three months after the echocardiogram appointment date (N, Y)
diff_surgery_after	The number of days between echocardiogram appointment was generated in the system and the surgery date (0–1, 2–5, 6–15, >=16, none)
Geo	Patient geo-location (InState, Town, OutState)
AGE	Age (0–18, 19–55, 56–65, 66–75, > 75)
Comorbidity	CHF	Comorbidities – congestive heart failure (N, Y)
	Valvular	Comorbidities – valvular disease (N, Y)
	PHTN	Comorbidities – pulmonary circulation disorders (N, Y)
	PVD	Comorbidities – peripheral vascular disease (N, Y)
	HTN	Comorbidities – hypertension (N, Y)
	Paralysis	Comorbidities – paralysis (N, Y)
	NeuroOther	Comorbidities – neurological disorders (N, Y)
	Pulmonary	Comorbidities – chronic pulmonary disease (N, Y)
	DM	Comorbidities – diabetes without chronic complications (N, Y)
	DMcx	Comorbidities – diabetes with chronic complications (N, Y)
	Hypothyroid	Comorbidities – hypothyroidism (N, Y)
	Renal	Comorbidities – renal failure (N, Y)
	Liver	Comorbidities – liver (N, Y)
	PUD	Comorbidities – chronic peptic ulcer (N, Y)
	HIV	Comorbidities – human immunodeficiency virus (N, Y)
	Lymphoma	Comorbidities – lymph system cancer (N, Y)
	Mets	Comorbidities – metastatic cancer (N, Y)
	Tumor	Comorbidities – solid tumor (N, Y)
	Rheumatic	Comorbidities – rheumatoid arthritis/collagen vascular (N, Y)
	Coagulopathy	Comorbidities – coagulation deficiency (N, Y)
	Obesity	Comorbidities – Obesity (N, Y)
	WeightLoss	Comorbidities – Weight Loss (N, Y)
	FluidsLytes	Comorbidities – fluid and electrolyte disorders (N, Y)
	BloodLoss	Comorbidities – blood loss (N, Y)
	Anemia	Comorbidities – anemia (N, Y)
	Alcohol	Comorbidities – alcohol abuse (N, Y)
	Drugs	Comorbidities – drug abuse (N, Y)
	Psychoses	Comorbidities – mental disorder characterized by a disconnection from reality (N, Y)
	Depression	Comorbidities – major depressive disorder (N, Y)
Referral diagnosis	A	MSSA bacteremia, sepsis
	B	MRSA, staph bacteremia, slaph, fungemia, pseudomonas, candidemia, MRSA bacteremia
	C	Leukemia, AML, cml, lymphoma, amy, myeloma
	D	Diseases of the blood and blood-forming organs and certain disorders involving the immune mechanism
	E	Endocrine, nutritional and metabolic diseases
	F	Behavioral and neurodevelopmental disorders
	G	Muscular dystrophy
	H	Diseases of the eye and adnexa or disease of the ear and mastoid process
	I	Heart failure, coronary artery, cardiac arrest, stemi, stroke, cardia, hypertension, endocarditis, ntemi, PEA arrest, fib, pulmonary embolism, pulm HTN, vegetation
	J	Resp failure, respiratory, pulmonary
	K	Liver, cirrhosis
	L	Diseases of the skin and subcutaneous tissue
	M	Diseases of the musculoskeletal system and connective tissue
	N	Diseases of the genitourinary system
	O	Pre-eclampsia, preeclampsia
	P	Certain conditions originating in the perinatal period
	Q	Ehlers, coarc, pda, congenital
	R	Murmur, hypox, shortness, SOB, breath, shock, dyspnea, chest pain, Troponin, Syncop, ekg, ecg, extremity, mass, swelling, edema
	S	Injury, poisoning and certain other consequences of external causes
	Z	Chemo, Preoperative, pre-op, prenatal, pregnancy, prior to, BMI, surgery, preop, transplant

## Methodology

In this work, we aim to apply the double machine learning technique to estimate the effects of various variables and provide insights for patient prioritization, while also accurately predicting appointment urgency. As a combination of statistical methods and machine learning techniques, double machine learning technique enables flexible modeling of the treatment assignment mechanism (i.e., the variable of interest whose effect on the outcome variable we seek to understand) and the outcome relationship. It is also capable of handling complex data and leveraging machine learning models with strong predictive power [7]. Double machine learning has been shown to be particularly effective in research settings where the treatment variable assignment is confounded by unobserved variables, and can improve the accuracy and precision of variable effect estimates compared to traditional methods such as regression-based methods or propensity score matching [8].

More specifically, double machine learning [7] entails a comprehensive framework designed to estimate effects of variables by harnessing machine learning techniques. The method first relies on a foundational framework to estimate variable effects, leveraging the inherent capabilities of machine learning algorithms to discern relationships within observational data sets. Notably, the estimation process incorporates the integration of confidence intervals, serving to elucidate the robustness and reliability of the derived estimates. Next, the methodology incorporates the utilization of an estimator characterized as “root n-consistent,” denoting its propensity to converge towards the true effect of variables (i.e., those with the greatest impact on outcome variable – patient urgency) as the sample size expands: this characteristic underscores the estimator’s favorable convergence attributes and data-efficiency, thereby enabling accurate estimation of variable effects even in scenarios with limited data availability. In essence, double machine learning epitomizes a methodical approach that integrates machine learning techniques, confidence intervals, and proficient estimators to derive dependable and insightful variable effect estimates.

In the case of effect identification of the echocardiography appointment urgence Y, there are N+1 variables ${W,X}_{\text{1}},\;X_{\text{2}},\;...,\;X_{N}\;$. These variables exhibit confounding relationships with one another (i.e., variables may influence one another while also directly impacting the outcome); for instance, *comorbidities* variables may exert influence on *diagnostic referrals* variables, while *administrative* variables may be contingent upon *diagnostic referrals* variables, meanwhile, all of these variables may exert influence on *appointment urgency*. Consequently, such interrelationships introduce complexities into the estimation of variable effects. Assume W is the variable of interest, the relationships of the variables can be characterized by a partial linear model [9]:


$$Y=W\theta_{\text{0}\;}+g_{\text{0}}(X)+U,\;E[U|\;W,X]=\text{0},$$ (1)

$$W=m_{\text{0}}(X)+V,\;E[V|X]=\text{0}.$$ (2)

where $X={(X}_{\text{1}},\;X_{\text{2}},\;...,\;X_{N})$, and *U* and *V* are disturbances or random noise. Equation (1) represents the primary equation, where $\theta_{\text{0}\;}$signifies the effect that is of interest to be estimated. Equation (2) is employed for tracking confounding by modeling the dependency of the treatment variable Won the covariates via the function $m_{\text{0}}(X)$. Double machine learning fits the functions $g_{\text{0}}(\cdot )$ and $m_{\text{0}}(\cdot )$ by machine learning models. In practice, double machine learning consists of three steps: 1) predict the treatment variable W by the other variables X; 2) predict outcome Y based on X; 3) regress the residuals from step 2) on the residuals from 1) and get an estimate of $\theta_{\text{0}\;}$. The prediction models used in steps 1) and 2) can be any machine learning model, providing flexibility of modeling different types of data. Double machine learning allows *W* to be a combination of different variables, which is a subset extracted from *X*. For each different *W*, Equation (2) is applied separately, thus yielding distinct effects of *W*.

## Experimental setting

In our research, aiming to estimate the unbiased effects of various clinical, administrative, and demographic variables on appointment urgency using double machine learning techniques, we categorize the variables into three groups: demographic variables, diagnosis variables, and comorbidity variables. For each iteration of our experiment, we use one of these variable groups (Demographic, Diagnosis, or Comorbidity) as the treatment variable W, and the remaining variables are used as covariates X. The treatment variable represents the set of variables whose effects we are interested in estimating, while the covariates represent the other factors that might influence both the treatment and the outcome.

For example, when estimating the effects of comorbidity variables, we use referral diagnoses and administrative variables as covariates. In each iteration, we apply a cross-validation procedure with 5-fold cross-validation with 10 repetitions. This allows for multiple splits of the data to evaluate model performance reliably, enhancing the model’s generalizability. Each split involves training the model on a portion of the data and testing it on the remaining portion, ensuring that predictions are not overfitted to specific subsets of the data.

## Results and discussion

In this section, we present three groups of experimental results. First, we identify the specific machine learning algorithm that achieves the best prediction performance in our research setting. The second experiment compares our double machine learning-based method with traditional machine learning methods in terms of prediction performance for patient urgency. The third experiment analyzes and discusses the estimated variable effects that can prioritize patient urgency.

### Selection of machine learning methods for double machine learning

For implementing double machine learning, the estimation of $g_{\text{0}}(\cdot )$ and $m_{\text{0}}(\cdot )$ involves selecting appropriate machine learning algorithms. Various choices of machine learning algorithms exist for this purpose. In this subsection, we present a comparison of results obtained by utilizing widely used machine learning algorithms in healthcare analytics [10]: Naive bayes [11], logistic regression [12], deep learning [13], decision tree [14], random forest [15], gradient boosting [16] and support vector machine (SVM) [17]. Note that $g_{\text{0}}(\cdot )$ and $m_{\text{0}}(\cdot )$ can be estimated by different machine learning models. However, for the sake of experimental simplicity, the same machine learning algorithms are employed for both $g_{\text{0}}(\cdot )$ and $m_{\text{0}}(\cdot )$ in this study.

In our experimental setup, we implemented a rigorous approach to model training and evaluation, adhering to established methodologies within the domain of machine learning. Employing a repeated cross-validation framework, we conducted three iterations of the experiments, each consisting of five-fold cross-validation. This experimental setup can ensure the robustness of our findings and to mitigate potential biases stemming from data partitioning of training and testing datasets. The evaluation metrics employed in this study include accuracy, precision, recall, and F1 score. Accuracy provides an assessment of the overall performance of the model, while F1 score, which is the harmonic mean of precision and recall, offers a robust evaluation measure particularly suitable for imbalanced datasets. The results are reported in Table 2.

**Table 2:** Double Machine Learning Implementation with Popular Machine Learning Methods.

Algorithm	Accuracy	Precision	Recall	F1
Naïve bayes	62.90(1.12)	33(0.78)	68.88(1.04)	44.61(0.74)
Weighted logistic regression	73.25(0.04)	42.87(0.05)	70.33(0.06)	53.27(0.04)
Deep learning	81.56(0.14)	63.42(1.05)	35.46(1.62)	45.45(1.18)
Decision tree	79.68(0.14)	58.85(0.82)	20.87(1.57)	30.78(1.72)
Random forest	82.15(0.05)	70.64(0.25)	30.23(0.22)	42.34(0.24)
Gradient boosting	**82.16(0.05)**	67.19(0.32)	34.62(0.21)	45.69(0.19)
SVM	82.12(0.03)	74.86(0.19)	26.4(0.13)	39.04(0.15)

According to the results, random forest, gradient boosting and SVM achieve the highest performance. SVM and weighted logistic regression outperform the others on accurately predicting urgent appointments and effectively identifying urgent cases in terms of precision and recall. Deep learning and gradient boosting demonstrate superior performance as indicated by their higher F1 scores, which suggests their overall reliability in the task. Considering its highest accuracy and F1 score among the evaluated models, gradient boosting proved to be a good choice for the subsequent evaluations.

In the context of predicting whether a patient is urgent or not, false negatives (missing urgent cases) can have severe implications for patients, potentially delaying necessary medical interventions and compromising patients’ health outcomes. Conversely, false alarms (incorrectly flagging non-urgent cases as urgent) may lead to increased workload and resource allocation for medical providers, potentially causing inefficiencies in the healthcare system. These considerations highlight the importance of selecting a model with high precision and recall, as well as a balance between minimizing false negatives and false positives; for this reason, gradient boosting is also an excellent choice for the subsequent analysis.

The double machine learning model using weighted logistic regression exhibits an accuracy of 73.25, which is relatively lower than the selected gradient boosting algorithm. However, it achieves an F1 score of 53.27, primarily driven by high recall, the highest among all the machine learning algorithms considered. To provide a comprehensive comparison, we present the estimated effects of each variable using the double machine learning model with logistic regression in Table A.2 in S1 Table, juxtaposed with the results from the double machine learning models implemented with gradient boosting in Table A.1 in S1 Table.

The weighted logistic regression double machine learning model places emphasis on minimizing false negatives and excels in detecting urgent cases. In Table A.2 in S1 Table, it is observed that all variables exhibit significance, indicating their usefulness in robustly identifying positive cases. Notably, *administrative* variables emerge with larger effects overall, suggesting its substantial influence in the prediction process.

Conversely, the double machine learning model implementing gradient boosting prioritizes overall performance, achieving higher accuracy but with a slightly lower F1 score compared to the logistic regression model. The detailed comparisons provided in the tables offer insights into the estimated effects of each variable under different modeling approaches. This analysis underscores the significance of both algorithms in their respective strengths: the weighted logistic regression model for its emphasis on detecting urgent cases with lower false negatives, and the gradient boosting double machine learning model for its comprehensive performance across the dataset.

### Appointment urgency prediction performance of double machine learning

One of our major goals is to accurately predict appointment urgency; it is imperative to verify the model’s efficacy through performance evaluation. In this section, we undertake a comparative analysis between the predictive outcomes derived from double machine learning and those generated by directly employing conventional machine learning algorithms.

Table 3 presents the evaluation metrics encompassing accuracy, precision, recall, and F1 score for each algorithm. Double machine learning exhibits a notable superiority in accuracy, achieving a rate of 82.16%, surpassing all the evaluated methods. It also achieved excellent recall performance (34.62%), outperforming other methods. Although its precision is lower than some of the benchmarks, it attained the highest F1 score, representing the harmonic mean of precision and recall, thus balancing prediction exactness and completeness. The results signify double machine learning’s adeptness in correctly identifying positive cases relevant to treatment variable effects, a critical aspect in diverse applications. In summary, double machine learning consistently exhibits superior performance in both accuracy and F1 score. The findings highlight that double machine learning, due to its ability to disentangle the complex relationships among variables and between variables and outcome variables, outperforms conventional machine learning methods in predicting appointment urgency within the context of our research.

**Table 3:** Double Machine Learning Performance Comparisons with Baselines.

Algorithm	Accuracy	Precision	Recall	F1
Naïve bayes	78.86(0.24)	81.3(7.11)	3.34(0.59)	6.41(1.09)
Weighted logistic regression	79.26(0.22)	77.68(4.26)	6.16(0.86)	11.41(1.49)
Deep learning	80.49(0.29)	85.59(4.59)	12.14(0.39)	21.26(0.66)
Decision tree	78.86(0.31)	80.07(9.49)	3.39(0.52)	6.50(0.99)
Random forest	79.45(0.18)	78.19(5.54)	7.34(0.31)	13.42(0.57)
Gradient boosted trees	80.64(0.29)	80.80(2.96)	14.94(1.55)	25.18(2.25)
SVM	80.30(0.84)	61.42(5.57)	24.06(3.4)	34.48(4.02)
Double machine learning	**82.16(0.05)**	67.19(0.32)	**34.62(0.21)**	**45.69(0.19)**

### Analysis of estimated effects of variables

**Fig 2:**
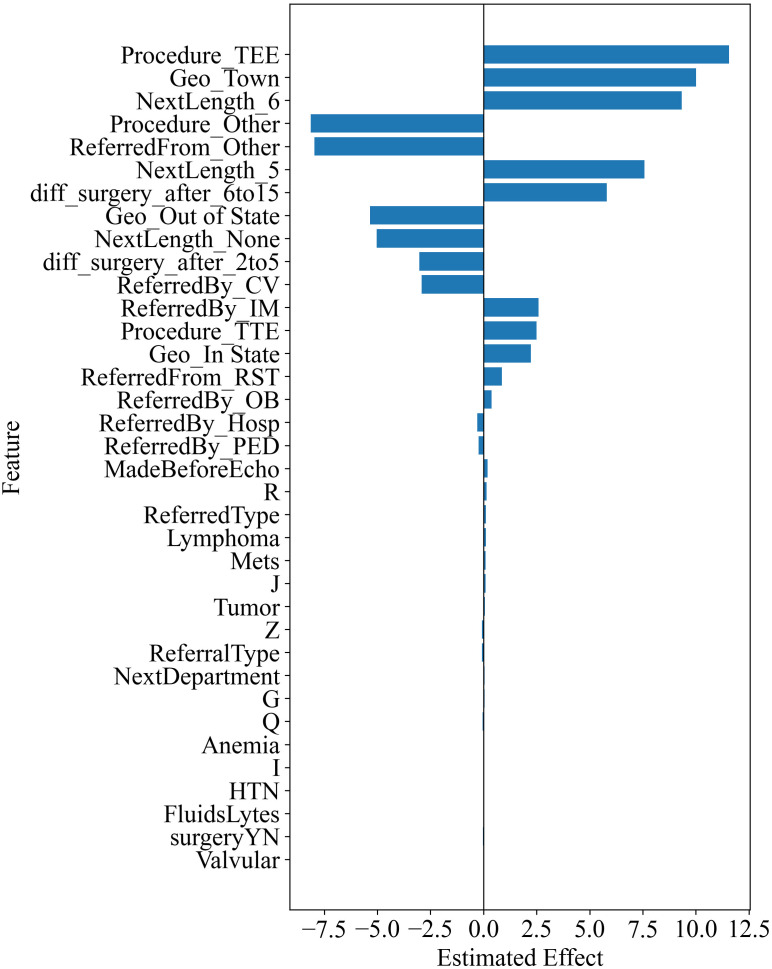
Estimated Variable Effects for Significant (P < 0.05) Variables from the Double Machine Learning Model.

Another important goal of this study is to estimate the effect of various clinical and administrative variables extracted from EHR data to identify and prioritize patients in need of echocardiography. In this subsection, we present the statistically significant variables, as indicated by a p-value less than 0.05, arranged by their level of significance (Table 4). To complement the numerical results, Fig 2 provides a visual summary of the estimated variable effects, illustrating the relative magnitude and direction of the most influential predictors across administrative, comorbidity, and referral diagnosis categories. The table containing all treatments alongside their respective statistical inference metrics, including standard deviation, t-value, p-value, and confidence intervals, is available in the S1 Table.

**Table 4:** Significant (P < 0.05) Variable Effects Estimated by Double Machine Learning Sorted by Significance in Each Category.

	Treatment	Coef
Administration	MadeBeforeEcho	0.19
	ReferredFrom_Other	−7.96
	NextDepartment	0.05
	ReferredType	0.12
	ReferredBy_CV	−2.92
	Procedure_Other	−8.14
	Geo_Town	10.01
	Geo_Out of State	−5.35
	NextLength_None	−5.03
	NextLength_5	7.58
	ReferralType	−0.07
	Procedure_TEE	11.57
	NextLength_6	9.33
	ReferredBy_IM	2.59
	Geo_In State	2.23
	diff_surgery_after_6–15	5.80
	ReferredBy_PED	−0.23
	surgeryYN	−0.03
	Procedure_TTE	2.50
	ReferredBy_OB	0.38
	ReferredBy_Hosp	−0.30
	ReferredFrom_RST	0.86
	diff_surgery_after_2–5	−3.02
Comorbidity	Lymphoma	0.11
	Mets	0.10
	Tumor	0.07
	HTN	0.03
	Valvular	−0.02
	Anemia	0.04
	FluidsLytes	0.03
Referral diagnosis	R	0.14
	Z	−0.07
	J	0.10
	I	0.04
	Q	−0.04
	G	0.05

The results in Table 4 and Fig 2 show that, of the total 36 significant variables, 23 are associated with *administration* variables, 7 pertain to *comorbidity* variables, and 6 relate to *referral diagnoses* variables. This observation suggests that many *administration variables* reflect prioritization of appointments. For instance, the most significant variable in this category, “MadeBeforeEcho,” denotes whether the subsequent downstream appointment following an echocardiogram is scheduled before the echocardiogram appointment was generated in the IT system. The significance and positive coefficient associated with this variable suggest that an affirmative status—where such proactive scheduling occurs—results in patients being given higher priority for their appointments. This finding aligns with intuitive expectations: healthcare providers often prioritize medical tests that are critical for informing or coordinating upcoming clinical appointments. Healthcare providers tend to expedite medical tests preceding upcoming appointments to ensure seamless coordination.

The three most significant *comorbidity* variables identified are all associated with cancer, underscoring a notable correlation between comorbidity patterns and oncological conditions. Existing research suggests that individuals diagnosed with cancer have diverse medical testing needs, often prioritizing aspects related to prognosis and a deeper understanding of their conditions [18]. The importance of cancer-related *comorbidity* variables align with established medical knowledge, emphasizing the heightened urgency for cancer patients to undergo echocardiographic assessments. Conversely, the top three *referral diagnoses* variable predominantly encompass cardiac and respiratory symptoms (R & J) or are linked to preparatory measures preceding medical interventions or surgical procedures (Z); the former directly pertains to cardiac ailments, while the latter signifies the imperative nature of patient assessments from a medical standpoint, notably preceding interventions or surgical interventions.

Among all the *administration* variables, transesophageal echocardiograms (TEE, Procedure_TEE in Table 4) emerge as the most impactful variable with a coefficient of 11.57. TEE, an invasive echocardiographic technique, is renowned for its superior diagnostic precision. It is commonly deployed when optimal visualization cannot be achieved via transthoracic echocardiograms (TTE), a limitation often attributable to impediments like scarring, excessive adipose tissue, or lung collapse. TEE’s diagnostic prowess proves pivotal, especially in identifying intricate cardiac pathologies such as atrial thrombi, infective endocarditis, aortic dissection, and select cardiac neoplasms. In scenarios where TTE renders inconclusive or suboptimal outcomes, TEE’s capacity to furnish intricate and contextually specific data promptly underscores its procedural urgency. Furthermore, TEE procedures frequently intersect with critical or unstable patient conditions, typified by suspected acute coronary syndromes, hemodynamic instability, or cardioembolic events. Despite representing only about 4% of all echocardiograms, the presence of TEE serves as a marker for these high-severity cases. This low proportion highlights its selective use in the most critical situations, thereby amplifying its overall impact on patient outcomes and treatment pathways. Timely execution of TEE facilitates expeditious evaluation of cardiac function and pathology, thereby informing emergent therapeutic interventions.

### Generalizability of findings

While our study demonstrates promising results in predicting appointment urgency based on clinical, administrative, and demographic variables, it is important to consider the generalizability of our findings to other settings. This study was conducted at a single center in Minnesota, which may not fully represent the diversity of healthcare systems, hospital sizes, and patient populations across the broader healthcare landscape.

The hospital in our study is a large multispecialty facility with a robust infrastructure and access to a wide range of healthcare services. Smaller hospitals or clinics may face different challenges, such as limited access to specialized diagnostic equipment or variations in staffing, which could affect the implementation and effectiveness of the model. Therefore, further validation of this model in hospitals with varying sizes and resource availability would be essential to assess its applicability in those settings.

Healthcare systems can vary significantly across regions, with differences in referral practices, appointment scheduling processes, and access to healthcare services. The model developed in this study is based on practices specific to the healthcare setting in Minnesota, and variations in how urgent appointments are determined and managed in other systems may limit the generalizability of the findings. For instance, in countries with universal healthcare systems or those with different models of care delivery, the treatment priorities and workflows may differ from those observed in this study.

To better understand the generalizability of our model, we suggest that future work should involve applying the framework to datasets from different hospitals and regions, including diverse patient populations and varying healthcare systems. Additionally, adapting the model to account for local healthcare conditions, policies, and workflows could increase its utility in broader settings. By doing so, the model could become a valuable tool for improving patient prioritization and resource allocation in diverse healthcare environments.

### Comparison with existing methods and advantages of double machine learning

While traditional methods such as regression models, propensity score matching, and logistic regression have been commonly used in healthcare for prioritization and predictive modeling, they have limitations in handling complex, high-dimensional data with multiple interdependent variables. Double machine learning offers an advantage by using machine learning models to predict the urgency, making it more effective at capturing the non-linear relationships and confounding variables that may be present in healthcare data. Traditional methods often fail to account for these interdependencies, which can lead to biased estimates and reduced prediction accuracy.

When compared to other advanced machine learning methods such as ensemble methods (e.g., XGBoost), random forest, support vector machines, and neural networks, double machine learning provides a clear advantage in terms of both prediction accuracy and interpretability. While these other methods often deliver robust predictions, they tend to function as “black boxes,” offering limited insight into how different variables influence the outcome. In contrast, double machine learning provides the added benefit of causal inference, allowing for more accurate effect estimation and variable importance analysis, which is critical in healthcare applications where understanding the impact of individual variables is crucial for clinical decision-making.

A significant advantage of the double machine learning approach is its ability to account for confounders and interdependencies among variables. In healthcare data, predictors like demographics, comorbidities, and clinical diagnoses are often correlated, making it challenging to isolate the true effect of one variable on the outcome. Double machine learning tackles this issue by first modeling the relationships between treatment and covariates, then using these models to adjust for confounding factors in the outcome model. This results in more robust and accurate estimates compared to traditional machine learning models that may overlook these complex relationships, making double machine learning particularly suitable for the multifaceted nature of healthcare data.

To further contextualize our approach, we compared the double machine learning framework with other advanced machine learning methods integrated into our experiments, including deep learning, random forest, gradient boosting, and support vector machines (Table 2). While these modern algorithms have demonstrated strong predictive power across many healthcare applications, they often operate as “black-box” systems that provide limited interpretability and may not adequately adjust for complex confounding relationships among variables. In contrast, double machine learning integrates these advanced learners within a causal inference framework, thereby combining the predictive strength of state-of-the-art algorithms with rigorous effect estimation. This hybrid structure enables the identification of unbiased variable effects and enhances clinical interpretability—an essential advantage when translating model outputs into actionable healthcare decisions.

Furthermore, we observed that while deep learning and ensemble models (e.g., gradient boosting and random forest) achieved competitive accuracy, the double machine learning approach consistently outperformed them in overall balance between accuracy and F1 score (Table 3), reflecting its ability to maintain predictive reliability while preserving interpretability. The framework’s flexibility allows different machine learning algorithms to serve as base learners, making it adaptable to future advancements in model architectures without compromising causal robustness. This demonstrates the scalability of double machine learning as a bridge between modern predictive analytics and transparent, clinically interpretable modeling for decision support in echocardiography prioritization.

### Clinical and operational implications

The findings from this study have several important clinical and operational implications that could directly impact the management of echocardiography appointments and patient care. Our urgency stratification model, which accurately predicts appointment urgency, can be integrated into existing clinical workflows to streamline the scheduling process, improve resource allocation, and enhance patient care delivery.

The model can help healthcare providers prioritize patients based on the urgency of their conditions, allowing for timely care for those with critical needs. By integrating the model into the hospital’s appointment scheduling system, staff can prioritize patients who require urgent echocardiograms, reducing the wait time for high-acuity patients. This system could prevent delays in diagnosis and treatment for patients with acute conditions, such as those suffering from severe heart failure or other life-threatening cardiac conditions.

The current scheduling system often faces challenges in managing high patient volumes and limited availability of resources. Our model can assist in optimizing the use of clinical resources, including sonographers, cardiologists, and available appointment slots. By identifying and scheduling urgent patients efficiently, the hospital can better allocate its human and physical resources to ensure that high-priority cases receive prompt attention. This could also reduce the occurrence of overbooking, a challenge that leads to inefficient use of resources and clinician burnout.

Timely scheduling of urgent appointments is likely to improve patient satisfaction by reducing unnecessary wait times for those needing immediate care. Moreover, by ensuring that the most critical cases are addressed promptly, the hospital can enhance its reputation for providing high-quality and responsive care. This could also result in better patient outcomes, as early diagnosis and treatment are crucial in many cardiovascular conditions.

## Conclusion

The echocardiogram laboratory serves as a pivotal hub for providing critical cardiac diagnostics, playing a vital role in managing various cardiovascular conditions. As echoed in the literature, echocardiography has emerged as a cornerstone in reducing mortality rates associated with cardiovascular diseases over recent decades. Despite its growing adoption, appointment prioritization remains challenging due to limited access to echocardiography laboratory services. Addressing these challenges requires a paradigm shift towards a comprehensive algorithmic approach that considers relevant clinical and administrative variables. In the digital age, machine learning methods offer promising avenues for enhancing the intelligence and capabilities of healthcare applications.

In this study, we leveraged double machine learning techniques to develop an appointment urgency stratification model aimed at prioritizing patients requiring echocardiography. Our methodology integrated clinical and administrative variables sourced from EHR data, allowing us to assess the effects of various variables and identify urgent appointments more effectively. Our analysis revealed the significant impact of certain variables pertaining to *administrative*, *comorbidity*, and *referral diagnoses* variables on patient prioritization. *Administrative* variables exhibit the highest effects. Cancer-related *comorbidity* variables surfaced as pivotal determinants of patient urgency, emphasizing the increased necessity for echocardiographic assessments among cancer patients. TEE emerged as a crucial diagnostic modality, providing superior precision in identifying complex cardiac pathologies, especially when TTE yield inconclusive results.

The experimental results highlight the potential of double machine learning in predicting appointment urgency and estimating variable effects robustly, consistently outperforming traditional methods across multiple evaluation metrics. By offering insights into the identification of urgent appointments and enhancing the estimate of variable effects, double machine learning holds promise for optimizing resource allocation in healthcare settings and improving patient outcomes.

The societal and economic benefits of this model are also noteworthy. By optimizing the scheduling process and accurately identifying urgent cases, the model can lead to significant cost savings by reducing overbooking, enhancing resource utilization, and preventing unnecessary delays in care. These improvements not only contribute to better patient outcomes but also help reduce the overall burden on healthcare systems, particularly in settings with limited resources. Additionally, the model’s ability to prioritize urgent cases more effectively can improve patient satisfaction by minimizing wait times and ensuring that patients with the most pressing needs receive timely care. This model can contribute to a more sustainable and equitable healthcare system, especially in resource-constrained environments, by optimizing the use of available resources and improving access to timely medical interventions.

Moving forward, the integration of advanced machine learning techniques with clinical decision-making processes presents exciting opportunities for transforming patient care and streamlining healthcare delivery. As we continue to refine and expand our understanding of the applications of double machine learning in healthcare analytics, we aim to contribute to the ongoing efforts aimed at enhancing the efficiency and effectiveness of cardiac diagnostic services.

## Supporting information

S1 Table Variable Effect Estimation by Double Machine Learning Model Using Gradient Boosting. (DOCX)
