# Supplementary material for: Unbiased inference for echocardiogram urgency prediction using double machine learning
Source: PLoS One. 2026 Jan 7;21(1):e0338922. doi: 10.1371/journal.pone.0338922 (PMC12779129; doi:10.1371/journal.pone.0338922)
Supplement: S1 Table — (DOCX) [file pone.0338922.s001.docx]

S1 Table. Variable Effect Estimation by Double Machine Learning Model Using Gradient Boosting

|  | Variable | Coef | Std err | t-value | P>\|t\| | 2.50% | 97.50% |
| --- | --- | --- | --- | --- | --- | --- | --- |
| Administration | MadeBeforeEcho | 0.1895 | 0.0099 | 19.2319 | 0.0000 | 0.1702 | 0.2088 |
|  | ReferredFrom_Other | -7.9645 | 0.7174 | -11.1026 | 0.0000 | -9.3705 | -6.5585 |
|  | NextDepartment | 0.0538 | 0.0051 | 10.5297 | 0.0000 | 0.0438 | 0.0638 |
|  | ReferredType | 0.1168 | 0.0114 | 10.2256 | 0.0000 | 0.0944 | 0.1392 |
|  | ReferredBy_CV | -2.9219 | 0.3173 | -9.2088 | 0.0000 | -3.5438 | -2.3000 |
|  | Procedure_Other | -8.1444 | 0.9852 | -8.2665 | 0.0000 | -10.0754 | -6.2134 |
|  | Geo_Town | 10.0069 | 1.2624 | 7.9266 | 0.0000 | 7.5326 | 12.4812 |
|  | Geo_Out of State | -5.3519 | 0.6910 | -7.7449 | 0.0000 | -6.7062 | -3.9975 |
|  | NextLength_0 | -5.0294 | 0.7641 | -6.5821 | 0.0000 | -6.5271 | -3.5318 |
|  | NextLength_5 | 7.5804 | 1.1754 | 6.4491 | 0.0000 | 5.2766 | 9.8841 |
|  | ReferralType | -0.0672 | 0.0119 | -5.6672 | 0.0000 | -0.0904 | -0.0440 |
|  | Procedure_TEE | 11.5691 | 2.3893 | 4.8420 | 0.0000 | 6.8861 | 16.2521 |
|  | NextLength_6 | 9.3276 | 2.1807 | 4.2773 | 0.0000 | 5.0534 | 13.6018 |
|  | ReferredBy_IM | 2.5929 | 0.7208 | 3.5971 | 0.0003 | 1.1801 | 4.0057 |
|  | Geo_In State | 2.2308 | 0.7041 | 3.1683 | 0.0015 | 0.8508 | 3.6109 |
|  | diff_surgery_after_6to15 | 5.8036 | 1.8840 | 3.0804 | 0.0021 | 2.1110 | 9.4962 |
|  | ReferredBy_PED | -0.2257 | 0.0809 | -2.7903 | 0.0053 | -0.3843 | -0.0672 |
|  | surgeryYN | -0.0252 | 0.0097 | -2.5851 | 0.0097 | -0.0443 | -0.0061 |
|  | Procedure_TTE | 2.4989 | 0.9940 | 2.5141 | 0.0119 | 0.5508 | 4.4470 |
|  | ReferredBy_OB | 0.3824 | 0.1713 | 2.2320 | 0.0256 | 0.0466 | 0.7181 |
|  | ReferredBy_Hosp | -0.3033 | 0.1433 | -2.1174 | 0.0342 | -0.5841 | -0.0226 |
|  | ReferredFrom_RST | 0.8571 | 0.4299 | 1.9935 | 0.0462 | 0.0144 | 1.6997 |
|  | diff_surgery_after_2to5 | -3.0205 | 1.5400 | -1.9614 | 0.0498 | -6.0388 | -0.0022 |
|  | NextLength_1 | -1.3016 | 0.9771 | -1.3321 | 0.1828 | -3.2166 | 0.6134 |
|  | GENDER_MALE | -0.0929 | 0.0941 | -0.9876 | 0.3233 | -0.2772 | 0.0915 |
|  | ReferredBy_FAM | 0.2096 | 0.2641 | 0.7935 | 0.4275 | -0.3081 | 0.7273 |
|  | diff_surgery_after_0-1 | -1.1578 | 1.6262 | -0.7120 | 0.4765 | -4.3452 | 2.0295 |
|  | AGE_56-65 | -0.0983 | 0.1429 | -0.6877 | 0.4916 | -0.3783 | 0.1818 |
|  | ReferredFrom_MCHS | -0.0347 | 0.0545 | -0.6374 | 0.5239 | -0.1415 | 0.0721 |
|  | GENDER_FEMALE | -0.0572 | 0.0966 | -0.5924 | 0.5536 | -0.2466 | 0.1321 |
|  | ReferredBy_Other | -0.0660 | 0.1785 | -0.3694 | 0.7118 | -0.4159 | 0.2840 |
|  | diff_surgery_after_>=16 | 0.2842 | 0.9400 | 0.3024 | 0.7624 | -1.5581 | 2.1265 |
|  | AGE_0-18 | -0.0437 | 0.1538 | -0.2843 | 0.7762 | -0.3451 | 0.2577 |
|  | AGE_66-75 | -0.0390 | 0.1452 | -0.2687 | 0.7881 | -0.3236 | 0.2455 |
|  | diff_surgery_after_None | -0.0817 | 0.7429 | -0.1100 | 0.9124 | -1.5378 | 1.3745 |
|  | SurgeryYN_After | 0.0538 | 0.7429 | 0.0724 | 0.9423 | -1.4024 | 1.5099 |
|  | AGE_>75 | 0.0094 | 0.1394 | 0.0676 | 0.9461 | -0.2637 | 0.2826 |
|  | AGE_19-55 | -0.0022 | 0.1532 | -0.0144 | 0.9885 | -0.3024 | 0.2980 |
| Comorbidity | Lymphoma | 0.1131 | 0.0193 | 5.8494 | 0.0000 | 0.0752 | 0.1510 |
|  | Mets | 0.1030 | 0.0247 | 4.1737 | 0.0000 | 0.0546 | 0.1514 |
|  | Tumor | 0.0655 | 0.0163 | 4.0255 | 0.0001 | 0.0336 | 0.0973 |
|  | HTN | 0.0348 | 0.0094 | 3.6970 | 0.0002 | 0.0163 | 0.0532 |
|  | Valvular | -0.0240 | 0.0072 | -3.3286 | 0.0009 | -0.0381 | -0.0099 |
|  | Anemia | 0.0366 | 0.0135 | 2.7114 | 0.0067 | 0.0101 | 0.0630 |
|  | FluidsLytes | 0.0335 | 0.0126 | 2.6655 | 0.0077 | 0.0089 | 0.0581 |
|  | WeightLoss | 0.0459 | 0.0240 | 1.9104 | 0.0561 | -0.0012 | 0.0930 |
|  | Rheumatic | 0.0498 | 0.0267 | 1.8642 | 0.0623 | -0.0026 | 0.1022 |
|  | Obesity | 0.0226 | 0.0130 | 1.7452 | 0.0810 | -0.0028 | 0.0480 |
|  | Pulmonary | 0.0251 | 0.0156 | 1.6072 | 0.1080 | -0.0055 | 0.0558 |
|  | PHTN | 0.0348 | 0.0221 | 1.5737 | 0.1156 | -0.0086 | 0.0782 |
|  | NeuroOther | 0.0232 | 0.0172 | 1.3441 | 0.1789 | -0.0106 | 0.0570 |
|  | Psychoses | 0.0493 | 0.0368 | 1.3394 | 0.1804 | -0.0228 | 0.1214 |
|  | DMcx | 0.0256 | 0.0229 | 1.1204 | 0.2625 | -0.0192 | 0.0704 |
|  | PVD | 0.0139 | 0.0125 | 1.1142 | 0.2652 | -0.0105 | 0.0383 |
|  | Drugs | -0.0471 | 0.0425 | -1.1067 | 0.2684 | -0.1305 | 0.0363 |
|  | Depression | 0.0186 | 0.0212 | 0.8789 | 0.3795 | -0.0229 | 0.0602 |
|  | Alcohol | 0.0267 | 0.0366 | 0.7295 | 0.4657 | -0.0450 | 0.0984 |
|  | Renal | 0.0066 | 0.0136 | 0.4839 | 0.6284 | -0.0200 | 0.0332 |
|  | PUD | -0.0138 | 0.0517 | -0.2671 | 0.7894 | -0.1151 | 0.0875 |
|  | DM | -0.0035 | 0.0169 | -0.2043 | 0.8381 | -0.0367 | 0.0297 |
|  | CHF | 0.0020 | 0.0100 | 0.1955 | 0.8450 | -0.0176 | 0.0215 |
|  | BloodLoss | -0.0054 | 0.0434 | -0.1248 | 0.9007 | -0.0906 | 0.0797 |
|  | Liver | -0.0024 | 0.0212 | -0.1147 | 0.9087 | -0.0441 | 0.0392 |
|  | Hypothyroid | 0.0013 | 0.0148 | 0.0853 | 0.9320 | -0.0277 | 0.0302 |
|  | Coagulopathy | 0.0013 | 0.0178 | 0.0753 | 0.9400 | -0.0335 | 0.0361 |
|  | Paralysis | 0.0011 | 0.0500 | 0.0216 | 0.9827 | -0.0970 | 0.0991 |
| Referral  diagnosis | R | 0.1414 | 0.0093 | 15.2228 | 0.0000 | 0.1232 | 0.1596 |
|  | Z | -0.0655 | 0.0073 | -8.9269 | 0.0000 | -0.0798 | -0.0511 |
|  | J | 0.0988 | 0.0190 | 5.2084 | 0.0000 | 0.0616 | 0.1360 |
|  | I | 0.0366 | 0.0073 | 4.9923 | 0.0000 | 0.0222 | 0.0510 |
|  | Q | -0.0435 | 0.0110 | -3.9369 | 0.0001 | -0.0651 | -0.0218 |
|  | G | 0.0459 | 0.0190 | 2.4187 | 0.0156 | 0.0087 | 0.0832 |
|  | L | 0.1266 | 0.0684 | 1.8508 | 0.0642 | -0.0075 | 0.2607 |
|  | F | 0.0947 | 0.0513 | 1.8462 | 0.0649 | -0.0058 | 0.1953 |
|  | E | -0.0176 | 0.0102 | -1.7191 | 0.0856 | -0.0377 | 0.0025 |
|  | M | 0.0280 | 0.0210 | 1.3344 | 0.1821 | -0.0131 | 0.0691 |
|  | K | -0.0308 | 0.0255 | -1.2069 | 0.2275 | -0.0808 | 0.0192 |
|  | B | 0.0518 | 0.0488 | 1.0597 | 0.2893 | -0.0440 | 0.1475 |
|  | N | 0.0155 | 0.0194 | 0.7959 | 0.4261 | -0.0226 | 0.0535 |
|  | T | -0.0229 | 0.0407 | -0.5638 | 0.5729 | -0.1026 | 0.0568 |
|  | O | -0.0208 | 0.0471 | -0.4411 | 0.6591 | -0.1132 | 0.0716 |
|  | D | 0.0066 | 0.0185 | 0.3580 | 0.7204 | -0.0297 | 0.0430 |
|  | A | -0.0173 | 0.0633 | -0.2735 | 0.7845 | -0.1413 | 0.1067 |
|  | P | 0.0087 | 0.0937 | 0.0927 | 0.9261 | -0.1749 | 0.1922 |
|  | C | -0.0012 | 0.0163 | -0.0723 | 0.9424 | -0.0331 | 0.0307 |
|  | H | 0.0015 | 0.0526 | 0.0289 | 0.9769 | -0.1015 | 0.1045 |

Table A.2. Variable Effect Estimation by Double Machine Learning Model Using Logistic Regression

|  | Variable | Coef | Std err | t-value | P>\|t\| | 2.5% | 97.5% |
| --- | --- | --- | --- | --- | --- | --- | --- |
| Administration | ReferralType | -0.3000 | 0.0058 | -51.4369 | 0.0000 | -0.3115 | -0.2886 |
|  | MadeBeforeEcho | 0.4551 | 0.0079 | 57.8230 | 0.0000 | 0.4397 | 0.4706 |
|  | NextLength_6 | 46.6418 | 0.8629 | 54.0541 | 0.0000 | 44.9506 | 48.3330 |
|  | Geo_Town | 61.1230 | 1.4352 | 42.5898 | 0.0000 | 58.3101 | 63.9358 |
|  | NextLength_5 | 25.9135 | 0.7207 | 35.9575 | 0.0000 | 24.5010 | 27.3260 |
|  | diff_surgery_after_6to15 | 18.9946 | 0.6051 | 31.3885 | 0.0000 | 17.8086 | 20.1807 |
|  | diff_surgery_after_2to5 | 18.1573 | 0.5916 | 30.6927 | 0.0000 | 16.9978 | 19.3168 |
|  | diff_surgery_after_0-1 | 17.5018 | 0.5854 | 29.8948 | 0.0000 | 16.3544 | 18.6493 |
|  | Procedure_TTE | -36.1281 | 1.3688 | -26.3933 | 0.0000 | -38.8110 | -33.4452 |
|  | surgeryYN | 0.1563 | 0.0062 | 25.1432 | 0.0000 | 0.1442 | 0.1685 |
|  | NextLength_1 | 21.9417 | 0.8903 | 24.6442 | 0.0000 | 20.1967 | 23.6867 |
|  | diff_surgery_after_>=16 | 13.0369 | 0.5958 | 21.8809 | 0.0000 | 11.8691 | 14.2047 |
|  | Procedure_TEE | 14.0626 | 0.8493 | 16.5575 | 0.0000 | 12.3979 | 15.7272 |
|  | Geo_In State | 20.0700 | 1.3364 | 15.0176 | 0.0000 | 17.4506 | 22.6893 |
|  | ReferredBy_IM | 5.6049 | 0.3773 | 14.8552 | 0.0000 | 4.8654 | 6.3444 |
|  | ReferredType | 0.1686 | 0.0139 | 12.1605 | 0.0000 | 0.1414 | 0.1957 |
|  | ReferredBy_CV | 3.3385 | 0.2848 | 11.7210 | 0.0000 | 2.7802 | 3.8968 |
|  | ReferredBy_FAM | 5.9310 | 0.5430 | 10.9232 | 0.0000 | 4.8668 | 6.9952 |
|  | Geo_Out of State | -12.5985 | 1.3080 | -9.6321 | 0.0000 | -15.1620 | -10.0349 |
|  | ReferredBy_OB | 4.9709 | 0.5480 | 9.0710 | 0.0000 | 3.8968 | 6.0449 |
|  | NextLength_0 | 8.9322 | 1.0039 | 8.8977 | 0.0000 | 6.9646 | 10.8998 |
|  | SurgeryYN_After | 25.2110 | 3.2598 | 7.7338 | 0.0000 | 18.8218 | 31.6001 |
|  | diff_surgery_after_None | -25.1746 | 3.2598 | -7.7228 | 0.0000 | -31.5637 | -18.7856 |
|  | Procedure_Other | 6.9481 | 1.0119 | 6.8664 | 0.0000 | 4.9648 | 8.9314 |
|  | ReferredBy_PED | 2.0974 | 0.3287 | 6.3811 | 0.0000 | 1.4532 | 2.7416 |
|  | ReferredBy_Other | -2.4222 | 0.3887 | -6.2311 | 0.0000 | -3.1841 | -1.6603 |
|  | AGE_0-18 | 1.8778 | 0.3141 | 5.9780 | 0.0000 | 1.2621 | 2.4935 |
|  | AGE_56-65 | 1.1420 | 0.2087 | 5.4713 | 0.0000 | 0.7329 | 1.5510 |
|  | AGE_>75 | 0.9299 | 0.1810 | 5.1366 | 0.0000 | 0.5751 | 1.2847 |
|  | AGE_19-55 | 1.0277 | 0.2014 | 5.1016 | 0.0000 | 0.6329 | 1.4225 |
|  | AGE_66-75 | 0.9075 | 0.1811 | 5.0123 | 0.0000 | 0.5527 | 1.2624 |
|  | ReferredFrom_Other | 1.2592 | 0.3402 | 3.7009 | 0.0002 | 0.5923 | 1.9260 |
|  | ReferredFrom_RST | -1.2154 | 0.5711 | -2.1280 | 0.0333 | -2.3348 | -0.0960 |
|  | ReferredFrom_MCHS | 1.9796 | 1.0208 | 1.9393 | 0.0525 | -0.0211 | 3.9804 |
|  | ReferredBy_Hosp | 0.3731 | 0.1988 | 1.8771 | 0.0605 | -0.0165 | 0.7627 |
|  | GENDER_FEMALE | 0.0752 | 0.1201 | 0.6265 | 0.5310 | -0.1601 | 0.3106 |
|  | NextDepartment | -0.0021 | 0.0061 | -0.3424 | 0.7320 | -0.0140 | 0.0099 |
|  | GENDER_MALE | -0.0092 | 0.1371 | -0.0672 | 0.9464 | -0.2779 | 0.2594 |
| Comorbidity | PHTN | 0.3103 | 0.0068 | 45.7935 | 0.0000 | 0.2971 | 0.3236 |
|  | HTN | 0.2599 | 0.0068 | 38.4014 | 0.0000 | 0.2466 | 0.2732 |
|  | NeuroOther | 0.3778 | 0.0072 | 52.6364 | 0.0000 | 0.3637 | 0.3919 |
|  | Pulmonary | 0.3452 | 0.0067 | 51.4991 | 0.0000 | 0.3320 | 0.3583 |
|  | DM | 0.3257 | 0.0071 | 45.9614 | 0.0000 | 0.3118 | 0.3395 |
|  | DMcx | 0.3272 | 0.0075 | 43.3619 | 0.0000 | 0.3124 | 0.3420 |
|  | Hypothyroid | 0.2908 | 0.0063 | 46.0671 | 0.0000 | 0.2785 | 0.3032 |
|  | Renal | 0.2859 | 0.0075 | 38.2168 | 0.0000 | 0.2712 | 0.3005 |
|  | Liver | 0.4063 | 0.0084 | 48.6562 | 0.0000 | 0.3899 | 0.4226 |
|  | Lymphoma | 0.4048 | 0.0087 | 46.3713 | 0.0000 | 0.3877 | 0.4219 |
|  | Mets | 0.4381 | 0.0095 | 46.2069 | 0.0000 | 0.4195 | 0.4566 |
|  | Tumor | 0.3578 | 0.0076 | 47.3466 | 0.0000 | 0.3430 | 0.3726 |
|  | Rheumatic | 0.3947 | 0.0071 | 55.6442 | 0.0000 | 0.3808 | 0.4086 |
|  | Coagulopathy | 0.3386 | 0.0080 | 42.3041 | 0.0000 | 0.3229 | 0.3543 |
|  | Obesity | 0.3021 | 0.0065 | 46.5259 | 0.0000 | 0.2893 | 0.3148 |
|  | WeightLoss | 0.5132 | 0.0089 | 57.3776 | 0.0000 | 0.4956 | 0.5307 |
|  | FluidsLytes | 0.3368 | 0.0082 | 41.2483 | 0.0000 | 0.3208 | 0.3528 |
|  | Anemia | 0.3795 | 0.0079 | 48.2539 | 0.0000 | 0.3641 | 0.3949 |
|  | Alcohol | 0.3808 | 0.0089 | 42.9591 | 0.0000 | 0.3634 | 0.3981 |
|  | Psychoses | 0.3730 | 0.0077 | 48.7150 | 0.0000 | 0.3580 | 0.3880 |
|  | Depression | 0.3573 | 0.0070 | 51.4064 | 0.0000 | 0.3437 | 0.3710 |
|  | PVD | 0.2293 | 0.0064 | 35.7201 | 0.0000 | 0.2167 | 0.2418 |
|  | BloodLoss | 0.2823 | 0.0080 | 35.2761 | 0.0000 | 0.2666 | 0.2980 |
|  | Paralysis | 0.3002 | 0.0088 | 34.1366 | 0.0000 | 0.2830 | 0.3174 |
|  | Drugs | 0.2924 | 0.0086 | 33.8829 | 0.0000 | 0.2755 | 0.3093 |
|  | CHF | 0.2013 | 0.0069 | 29.0882 | 0.0000 | 0.1877 | 0.2149 |
|  | PUD | 0.2697 | 0.0098 | 27.3823 | 0.0000 | 0.2504 | 0.2890 |
|  | Valvular | 0.1254 | 0.0060 | 20.9252 | 0.0000 | 0.1136 | 0.1371 |
| Referral  diagnosis | A | 0.4571 | 0.0115 | 39.8248 | 0.0000 | 0.4346 | 0.4796 |
|  | B | 0.3638 | 0.0086 | 42.2530 | 0.0000 | 0.3469 | 0.3806 |
|  | D | 0.3021 | 0.0063 | 48.1480 | 0.0000 | 0.2898 | 0.3144 |
|  | E | 0.2282 | 0.0059 | 38.3959 | 0.0000 | 0.2166 | 0.2399 |
|  | F | 0.4535 | 0.0091 | 49.7862 | 0.0000 | 0.4357 | 0.4714 |
|  | G | 0.3460 | 0.0070 | 49.5637 | 0.0000 | 0.3323 | 0.3597 |
|  | H | 0.4352 | 0.0099 | 43.8034 | 0.0000 | 0.4157 | 0.4546 |
|  | J | 0.4218 | 0.0072 | 58.8357 | 0.0000 | 0.4078 | 0.4359 |
|  | K | 0.3611 | 0.0081 | 44.5565 | 0.0000 | 0.3452 | 0.3770 |
|  | M | 0.4001 | 0.0074 | 54.3436 | 0.0000 | 0.3856 | 0.4145 |
|  | N | 0.2715 | 0.0067 | 40.4490 | 0.0000 | 0.2584 | 0.2847 |
|  | R | 0.3078 | 0.0075 | 41.2920 | 0.0000 | 0.2932 | 0.3224 |
|  | L | 0.3426 | 0.0092 | 37.0955 | 0.0000 | 0.3245 | 0.3607 |
|  | C | 0.3504 | 0.0096 | 36.6434 | 0.0000 | 0.3316 | 0.3691 |
|  | T | 0.2305 | 0.0070 | 33.1595 | 0.0000 | 0.2169 | 0.2441 |
|  | Z | 0.1328 | 0.0066 | 20.1387 | 0.0000 | 0.1199 | 0.1457 |
|  | Q | 0.1550 | 0.0081 | 19.2189 | 0.0000 | 0.1392 | 0.1708 |
|  | O | 0.2573 | 0.0165 | 15.6196 | 0.0000 | 0.2250 | 0.2896 |
|  | P | 0.1771 | 0.0289 | 6.1353 | 0.0000 | 0.1205 | 0.2336 |
|  | I | 0.0346 | 0.0080 | 4.3105 | 0.0000 | 0.0189 | 0.0503 |
